# **FLUTE:** Fast and reliable knowledge retrieval from biomedical literature

**DOI:** 10.1093/database/baaa056

**Published:** 2020-08-06

**Authors:** Emilee Holtzapple, Cheryl A Telmer, Natasa Miskov-Zivanov

**Affiliations:** 1 Department of Computational and Systems Biology, University of Pittsburgh, 3501 Fifth Ave, Pittsburgh, Pennsylvania 15213, USA; 2 Molecular Biosensor and Imagining Center, Carnegie Mellon University, 4400 Fifth Ave, Pittsburgh, Pennsylvania 15213, USA; 3 Department of Electrical and Computer Engineering, University of Pittsburgh, 3700 O’Hara St, Pittsburgh, Pennsylvania 15261, USA; 4 Department of Bioengineering, University of Pittsburgh, 300 Technology Dr, Pittsburgh 15213, USA

## Abstract

State-of-the-art machine reading methods extract, in hours, hundreds of thousands of events from the biomedical literature. However, many of the extracted biomolecular interactions are incorrect or not relevant for computational modeling of a system of interest. Therefore, rapid, automated methods are required to filter and select accurate and useful information. The FiLter for Understanding True Events (FLUTE) tool uses public protein interaction databases to filter interactions that have been extracted by machines from databases such as PubMed and score them for accuracy. Confidence in the interactions allows for rapid and accurate model assembly. As our results show, FLUTE can reliably determine the confidence in the biomolecular interactions extracted by fast machine readers and at the same time provide a speedup in interaction filtering by three orders of magnitude.

Database URL: https://bitbucket.org/biodesignlab/flute.

## Introduction

The amount of published material produced by experimental laboratories is increasing at an incredible rate, limiting the effectiveness of manually analyzing all available information. And yet, there is a wealth of information in published articles so that if automated methods were developed to gather and extract the vast knowledge present in the literature, coupled with automated assembly of computational models, it would have a great impact on the understanding of large complex systems. State-of-the-art natural language processing (NLP) can rapidly ‘read’ very large amounts of biomedical literature, which reduces the amount of time needed to extract specific information from text (1, 2).

In the NLP-based machine reading, cellular event extraction relies on algorithms that recognize patterns in human written text, which allows for rapid processing of biomedical literature and for the extraction of information about biochemical interactions. Moreover, event extraction is distinguishable from text mining in that it not only recognizes keywords but also can retrieve the context from the literature. Since biomolecular interactions usually include two (or more) entities and an influence between them, event extraction also provides information about the direction (i.e. regulator and regulated element) and sign (i.e. positive or negative regulation) of molecular interactions. Recently, Integrated Network and Dynamical Reasoning Assembler (INDRA) (3) has been proposed to efficiently utilize machine readers (4–6) for cellular event extraction from selected literature. In biomedical literature, these entities are proteins, genes, chemicals and biological processes. The interaction between them may be a post-translational modification, transcriptional regulation, complex formation, etc. It is important to note that not all interactions represent a physical contact between entities (direct interaction). Instead, the influence may be propagated through one or more intermediaries (indirect interaction). While the direction and the sign of interaction are both output by INDRA, there is no indication of whether an interaction is direct or indirect. On the other hand, the Dynamic System Explanation (DySE) framework (7) uses both direct and indirect interactions obtained by machine reading to automatically assemble executable models at different levels of abstraction. DySE conducts automated model testing before using models to explain systems, predict system behavior, or guide interventions; however, the accuracy or confidence in its output depends on the correctness or confidence that we have in the automatically extracted interactions.

Obtaining reliable information about biochemical interactions is necessary for automated creation, testing and use of computational models. However, machine-based event extraction is still prone to error, requiring human intervention to correct the output obtained automatically. There are several types of errors that are encountered in machine reading output resulting from ambiguous natural language descriptions of complex biological events such as complex formation and further ambiguities involving protein families and biomolecule name aliases. Therefore, the validation of machine reading results is critical for downstream modeling, as well as for further improvements of event extraction itself.

To validate the results of event extraction from biomedical literature, a variety of methods have been used. The uncertainty within the language describing biomolecular events can be used to score individual interactions (8). Methods using human annotation to validate findings of machine reading have also been demonstrated (9). However, these validation methods focus on improving existing tools, which is beneficial, but they do not provide scoring or filtering for immediate use. While the field of NLP research continues to grow and improve, additional validation is still needed for extracted events in order to build reliable models.

Public databases like the Search Tool for the Retrieval of Interacting Genes and Proteins (STRING) (10–12) include curated information about protein–protein interactions (PPIs), which is frequently used for reconstruction of PPI networks (13) or for scoring individual interactions (14). Other databases such as the Gene Ontology (GO) Consortium (15, 16) maintain annotations on genes and associated biological processes. While machine reading methods allow for the retrieval of cell-type-, tissue- or disease-specific results, existing interaction databases often do not have this contextual information. Therefore, retrieving all potentially relevant interactions from databases may not match the context of the system of interest and would need to be experimentally verified. Additionally, machine reading can extract novel interactions that have not been added to interaction databases. Still, due to the complexity of the systems being studied and the language describing the interactions, the accuracy of machine reading can vary, with many methods for event extraction in biomedical literature having less than 70% precision (17).

By applying a filter to the cellular events extracted using machine reading, automatically assembling models will be more accurate and less computationally expensive. Here, we propose the FiLter for Understanding True Events (FLUTE) to select interactions with high confidence from the set of events extracted by machine reading. The main contributions of the proposed work are as follows:

(i) a fast automated tool to ‘reduce’ the vast number of cellular events extracted by machine reading and to facilitate ‘rapid’ model building;
(ii) a filtration methodology to ‘select’ interactions for addition to an existing model and to ‘increase confidence’ in the interactions added to the model.

## Methodology

**Figure 1:**
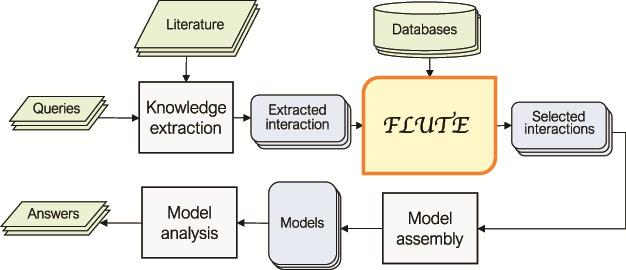
An outline of the role of FLUTE in the automated information extraction and the model assembly and analysis flow: FLUTE uses available databases to filter extracted interactions obtained as output of knowledge extraction process from available literature, which is usually initiated by user queries. The selected interactions from FLUTE are inputs to model assembly that creates models, which are then explored with model analysis in order to provide answers to user questions.

In Figure 1, we outline a typical FLUTE workflow. The selection of inputs for FLUTE is guided by user queries and can be compiled, through manual or machine reading of literature, into a set of ‘extracted interactions’. While FLUTE is best utilized when filtering machine reading output, it can be applied to manually extracted interactions as well. FLUTE outputs ‘selected interactions’, a subset of extracted interactions, which can then be used to assemble models and help answer the queries. In the following subsections, we provide the details of our approach to collect inputs for FLUTE and the FLUTE filter implementation.

### The preprocessing of FLUTE inputs

The input format used by FLUTE is shown in Table 1. FLUTE relies on a tabular event representation format, where the unique identifiers (IDs) for both participants are known. The element types (protein, biological process, etc.) can be inferred from the ID. While FLUTE does not explicitly check the effect of the interaction, or the reference listed, this information can be used by human curators or by downstream model assembly tools. Each extracted interaction also has an associated evidence statement with the text from which the event was extracted and could also be used for human judgment.

**Table 1:** Sample input to FLUTE

Regulated	Regulated type	Regulated ID	Regulator	Regulator type	Regulator ID	Effect	Reference	Evidence (sentence segment)
apoptosis	biological process	GO:0006915	Fas	protein	P25445	increases	PMC149420	‘Cell lines resistant to Fas mediated apoptosis…’
beta-catenin	protein	P35222	Axin	protein	O15169	decreases	PMC4102778	‘A complex is formed consisting of and APC, Axin, beta-catenin …’
GSK-3beta	protein	P49841	sorafenib	chemical	216 239	increases	PMC4102778	‘The multi-kinase inhibitor sorafenib induced GSK-3beta…’

Each row corresponds to one event extracted from literature using machine reading.

The type of the regulator and regulated element, along with the standardized IDs, are used to find support in databases.

FLUTE can process any set of interactions, from any source, as long as the interactions are written in the FLUTE input format outlined in Table 1. As stated earlier, using FLUTE becomes especially critical when the number of extracted interactions is of the order of several hundred or more, and therefore, not practical for manual filtration. Fast machine reading attempts to comprehensively extract relevant information from scientific literature, thus most often returning hundreds or thousands of interactions. For example, following the flow outlined in Figure 1, we can type into a literature search engine (e.g. PubMed (18)) a query written as a logical expression:

(1) \begin{equation*} \textit{T-cell}\ \mathbf{A}\mathbf{N}\mathbf{D}\ \left(\textit{PTEN}\ \mathbf{O}\mathbf{R}\ \textit{AKT}\ \mathbf{O}\mathbf{R}\ \textit{FOXO}\right). \end{equation*}

For the query above, PubMed returns relevant papers where *T-cell* is mentioned together with either *PTEN* or *AKT* or *FOXO*. Next, machine reading engines extract events from the papers listed by PubMed*.* For well-studied pathways and molecules, queries can result in a large number of papers found in PubMed and, consequently, a large number of interactions. We investigated the influence of query categories and topics on the number of papers found, and on the percentage of selected interactions output by FLUTE, and we discuss our findings in Results - Influence of query choice.

**Figure 2:**
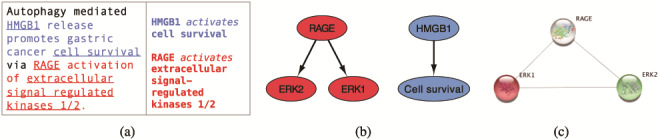
From sentences to scored interactions: (a) example sentence and the corresponding machine reading output; (b) graphical representation of interactions; (c) interactions found between ERK1, ERK2 and RAGE by STRING.

We use the INDRA framework and the reading engine REACH (6, 19) to extract relevant information from selected literature. In Figure 2a, we show an example sentence from a scientific text (20) and the corresponding REACH output for this sentence. The three interactions that the reading engine was able to extract from this sentence can be visualized as a graph, shown in Figure 2b, where the interacting elements are represented as nodes and the interactions as directed edges. Searching the STRING public database for interactions between elements that machine reading has extracted (ERK1, ERK2 and RAGE), returns a graph shown in Figure 2c.

Through manual curation of machine reading output, we identified four major types of errors in the extracted interactions, namely ambiguous or misconstrued sentences (Omission error), interactions where one or both elements are incorrectly grounded (Grounding error) and interactions that have opposite directionality (Direction error) or opposite effect (Sign error) (7). In the case of Omission error, the reader denotes a relationship between two elements that does not exist in the evidence statement, while in the Grounding error, the reader was unable to match the elements in the interaction to the correct IDs. As an example, for the evidence statement ‘Although Tcf3 binds GSK3, it does not inhibit the activity of GSK3 against axin.’, machine reading gives us the following interaction: ‘Tcf3 inhibits GSK3’. Due to the verbosity of the sentence, machines output an incorrect interaction. From the sentence ‘CtIP (CtBP interacting protein) is also critical for HR mediated DSB repair’, machine readers extract an interaction where HR regulates DSB, both classified as proteins. However, DSB stands for double-stranded break, not the protein DSB, thus leading to a grounding error. We have explored the distribution of these error types in the context of several queries, and we describe our findings in Results - Influence of machine reading errors.

### Implementation of FLUTE

**Figure 3:**
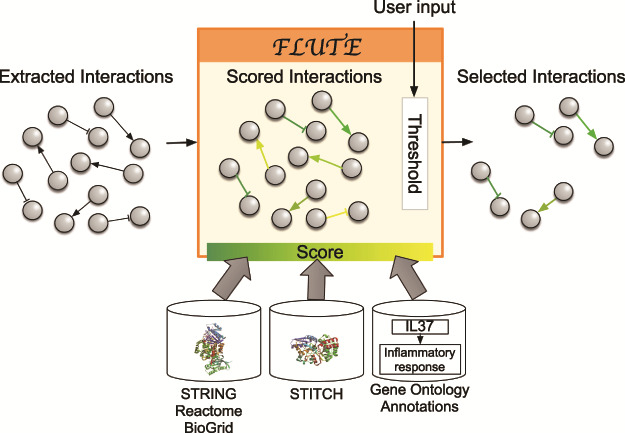
Filtration process with FLUTE: inputs to FLUTE include extracted interactions, scores of these interactions that are found in databases, and the user’s selection of thresholds for the scores. Outputs from FLUTE include selected interactions determined by their scores and thresholds.

The goal of the filtration procedure would ideally be to select accurate interactions from the collected machine reading output. However, such reliable filtering of the machine reading output is difficult due to the errors that are common in this output (as discussed above). To validate the accuracy of machine reading output, interactions could be compared to the set of all known human protein–protein, protein–biological process or protein–chemical interactions, but the number of PPIs alone is somewhere in the vicinity of 15 million and is expanding rapidly. The existing interaction databases (12, 16, 21–23) contain manually curated interactions and are supported by multiple paper sources and experimental evidence such as co-expression and co-immunoprecipitation assays. Still, the databases often do not include the information about context, direction and sign of interactions. To harness the advantages of both query-specific machine reading and more reliable databases, FLUTE uses the databases to determine the confidence in the interactions extracted from literature by machines. Using established interaction databases together with machine reading of published literature will enable assembling more realistic models of biological systems (Figure 1). We illustrate in Figure 3 the key components participating in the FLUTE interaction filtering.

In biomedical literature, descriptions of intracellular interactions can include different types of molecules and biological phenomena. Table 2 lists all the element types that can participate in interactions extracted by machines, as well as databases used to determine the unique IDs of elements. Using unique IDs is necessary for machines to be able to quickly pass information between different software tools (e.g. from machine readers to FLUTE, or from FLUTE to model assembly). Furthermore, in this work, we focused in particular on developing a filter for several types of interactions: (i) PPIs, (ii) protein-biological process interactions (PBPIs) and (iii) protein-chemical interactions (PCIs). The databases we used are the GO Annotation (15, 16), STITCH (21), BioGrid (22), Reactome (23, 24) and STRING (10–12) databases.

The STRING database contains information about the presence and confidence of predicted PPIs. STRING curates several different types of data on PPIs such as physical interactions, homologous sequences and co-mentions in databases. The interactions in STRING are drawn from pre-existing databases or manually extracted from either whole manuscripts or abstracts. STRING also scores the confidence in an interaction as a numeric value from 0 (low confidence) to 1000 (high confidence). Furthermore, there is detailed information on association type available for a subset of interactions. We also incorporated two other PPI databases, Reactome and BioGrid. While these databases store the same type of interactions as STRING, they are smaller and do not have scoring metrics. STITCH is a sister database to STRING and can be used in the same manner for PCIs. GO annotations can be used to judge the quality of gene-biological process interactions. A GO term is a functional association between a gene and a cellular process, and the GO annotations are based upon several different evidence types and are subject to multiple quality control measures (25).

**Table 2:** The different types of elements in machine reading output and databases used to assign unique identifiers to elements of these types

Element type	Databases
Biological process	GO (15, 16)
Protein	UniProt (26)
Chemical	PubChem (34)
Protein family	PFAM (30), InterPro (35)

**Figure 4:**
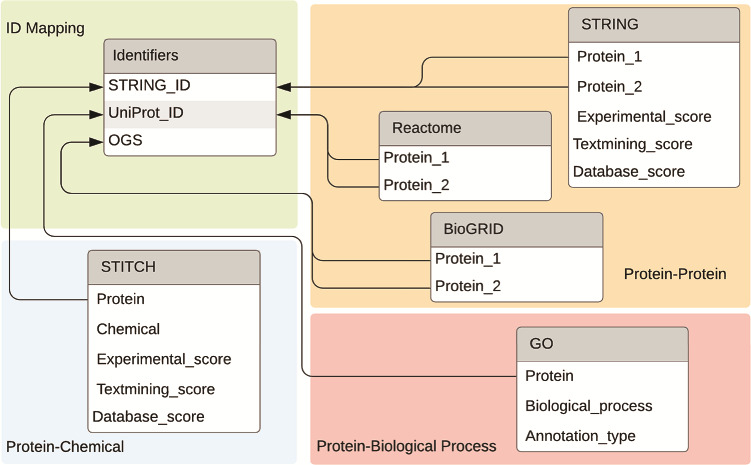
Databases and the connections between databases used by FLUTE.

For ease of use, we built a MySQL database and stored the interaction information offline in this database (https://bitbucket.org/biodesignlab/flute). The database schema is shown in Figure 4. The database contains six tables total, which can be classified into four categories: PPI data, PCI data, PBP data and ID mapping. The aggregated FLUTE database contains more than 30 million unique interactions. This setup is easily utilized to select multiple interaction types from the reading, based on the level of support found in the literature. We imported PPI data from STRING, BioGRID and Reactome. STRING, which is a larger database, also contains scores for the confidence of the interaction. Experimental evidence that shows physical binding increases the experimental score (escore). The database score (dscore) is derived from curated data from other sources. The textmining score (tscore) measures the co-occurrence of the two proteins in abstracts. The STRING scoring algorithm is described in greater detail in (12). These fields are present for all PPIs in the STRING database. The other score types include co-expression, homology, fusion, phylogeny and neighborhood scores. However, these are less likely to have nonzero values and, as such, these were not implemented in the FLUTE database. In contrast to STRING, Reactome and BioGRID PPI information does not contain a score, and therefore, it was incorporated in FLUTE simply as an indication of whether an interaction is known. All PCI data is imported from STITCH. As the sister database of STRING, the escore, tscore and dscore from STITCH are computed similarly. We also incorporated in our MySQL database a list of all GO annotations. While there is no ‘score’ for the confidence of these annotations, there is an annotation type that describes the curation method (e.g. experimentally, electronically, etc.).

The final table included in the FLUTE database schema is a mapping for all STRING IDs to their UniProt (26) IDs and official gene symbol (OGS). Grounding is an important step in the machine reading process, as it assigns a unique ID to each extracted entity. While STRING and STITCH use Ensembl IDs for proteins, Reactome and GO use UniProt IDs. BioGRID uses OGS, instead of either of the previously mentioned ID types. Figure 4 shows the relationships between the ID table and fields that can be converted. While the ID mapping table contains all known data for each of the three ID types, there is no guarantee that all three fields will be available for every known protein.

Dynamic models assembled downstream of FLUTE require the information about interaction direction; therefore, it is important to note that STRING does not always include a direction in the interactions it supports. Therefore, FLUTE obtains this information from the machine reading output. If there is a specific interaction, for example, phosphorylation, that is clearly directed in STRING. Similarly, STRING can determine if an interaction is positive or negative depending on whether there is evidence for the sign of interaction. However, this information is not always available, and so it has not been implemented in FLUTE, that is, if an extracted interaction matches the available data, it will be selected, even if the interaction has a Sign error.

#### *Using FLUTE database thresholds to select interactions from the extracted interactions set*.

When a new input from machine readers is provided to FLUTE in response to a user query, FLUTE matches elements within this input to the information in the ID mapping table. Once all IDs have been matched, FLUTE searches the relevant databases for each interaction. For example, the search for PPIs is conducted on the Reactome, BioGRID and STRING databases. All supporting fields, such as scores, are extracted and reported for all interactions. FLUTE discards any unmatched interactions. Furthermore, besides guiding literature selection with queries, FLUTE allows users to select ‘database thresholds’, that is, interaction score thresholds that tailor the number and confidence of the selected interactions. For each interaction type, FLUTE can select only interactions that meet a certain score. For example, FLUTE can return only PPIs and PCIs with an escore of >0, which guarantees that all selected interactions have at least one source of experimental data. A higher score threshold will decrease the number of selected interactions, but it will also increase the confidence in the selected interactions.

**Table 3:** Queries: different topic categories, example query terms for each topic category and the corresponding query expressions entered in PubMed

#	Query topic category	Query terms	Query expression
1	Disease	Breast cancer	‘breast cancer’
2	Cellular process	DNA repair	‘DNA repair’
3	Signaling pathway	MAPK/ERK pathway	‘erk pathway’ or ‘mapk pathway’ or ‘ras pathway’
4	Protein	BRCA1	BRCA1
5	Chemical	Progesterone	Progesterone
6a	Disease and process	Breast cancer, DNA repair	‘breast cancer’ and ‘dna repair’
6b		Autophagy, cancer	autophagy and cancer
7	Disease and pathway	Breast cancer, MAPK/ERK pathway	‘breast cancer’ and (‘erk pathway’ or ‘mapk pathway’ or ‘ras pathway’)
8	Disease and protein	Breast cancer, BRCA1	‘breast cancer’ and brca1
9	Disease and chemical	Breast cancer, progesterone	‘breast cancer’ and progesterone
10a	Process and protein	DNA repair, BRCA1	‘dna repair’ and brca1
10b		ADAM17, inflammation	ADAM17 and inflammation
11a	Well studied	EGFR	EGFR
11b		HER2	her2
12	New discovery	copb2*	copb2
13a	Multiple aliases	RSK90	‘rsk 90’
13b			RPS6KA1
13c			RSK-1
13d			S6K
14a	Non-standard characters	Beta catenin	CTNNB1
14b			‘Beta catenin’
14c			Beta-catenin
14d			CTNNB
15a	Different gene and	Estrogen receptor	‘Estrogen Receptor 1’
15b	Protein name		ESR1
15c			ER
16a	Same gene and	PTEN	Pten
16b	Protein name	GRB2	GRB2

#### *Using non-database thresholds to select interactions from the extracted interactions set*.

To complement the selection that relies on database thresholds, FLUTE can also select interactions based on the year of publication and their repeated occurrence in literature. We define two ‘non-database thresholds’: one threshold for the earliest allowed publication year, and another threshold for the least required number of papers that mention the same interaction. Following these thresholds, FLUTE can flag interactions from recently published papers as potentially novel interactions and interactions that appear in multiple papers as between-paper duplicates. Besides the between-paper duplicates, there are also within-paper duplicates, that is, interactions repeating in the same paper. However, if interactions are repeated in one paper only, we assume that they are lower-confidence interactions when compared to those that appear in multiple papers, and therefore, we did not implement an optional flag for the within-paper duplicates. By marking interactions as potentially novel or between-paper duplicates, FLUTE allows the user, if desired, to find the interactions that have been recently published, or those that have more support in the literature. For example, a user query for a well-known pathway may include a gene or protein with a recently discovered function. In this case, interaction databases may not be up-to-date and the option to select potentially novel interactions or between-paper duplicates could be beneficial for modeling. Furthermore, since these interactions are flagged, the user can easily find them and conduct a further manual review.

#### *Using FLUTE database to select additional database interactions*.

Besides selecting high-confidence interactions using database thresholds, the FLUTE database can also be utilized to find interactions by their citation. In other words, to supplement the results of machine reading, we implemented a function in FLUTE that searches the FLUTE database for additional interactions that cite the same papers as those read by reading engines and includes this set of interactions in the output. This FLUTE function allows for finding interactions in the selected papers that reading engines have missed.

#### Using FLUTE database to select relevant literature.

Different from the FLUTE flow described previously, which starts with searching for relevant papers in PubMed, FLUTE is also able to search the interaction databases to find relevant papers. This utility would solve a very common issue that is observed when the papers are retrieved from PubMed, namely, finding papers that contain no recognizable signaling events. Following a search query that includes one or more proteins, the FLUTE paper retrieval function searches for open-access papers cited by interactions in databases that mention at least one interaction involving at least one of the query proteins. Once the papers are found, the remaining steps are the same as already described in the paper, i.e. machine readers extract interactions from the papers, and FLUTE then filters these interactions.

## Results

### Influence of query choice

To explore the influence of various topics that could be included in queries, we obtained FLUTE results for 28 different queries (Table 3). We explored 16 different query topic categories (e.g., ‘Disease and Pathway’, Q7), then we chose example topic for each category (e.g., ‘Breast Cancer, MAPK/ERK pathway’), and finally the terms for each query topic that are combined into a machine readable query written as a logical expression (e.g., ‘*breast cancer*’ **AND** (‘*Erk pathway*’ **OR** ‘*MAPK pathway*’ **OR** ‘*Ras pathway*’). In these exercises, we also accounted for the fact that, in biological literature, different aliases can be used across biological papers to represent the same entity (e.g. ‘rsk 90’ or RPS6KA1 or RSK-1 or S6K, in Q13a-d), and that some aliases include characters that are not accurately recognized by machine reading engines (e.g. ‘-’ in Q13c).

**Figure 5:**
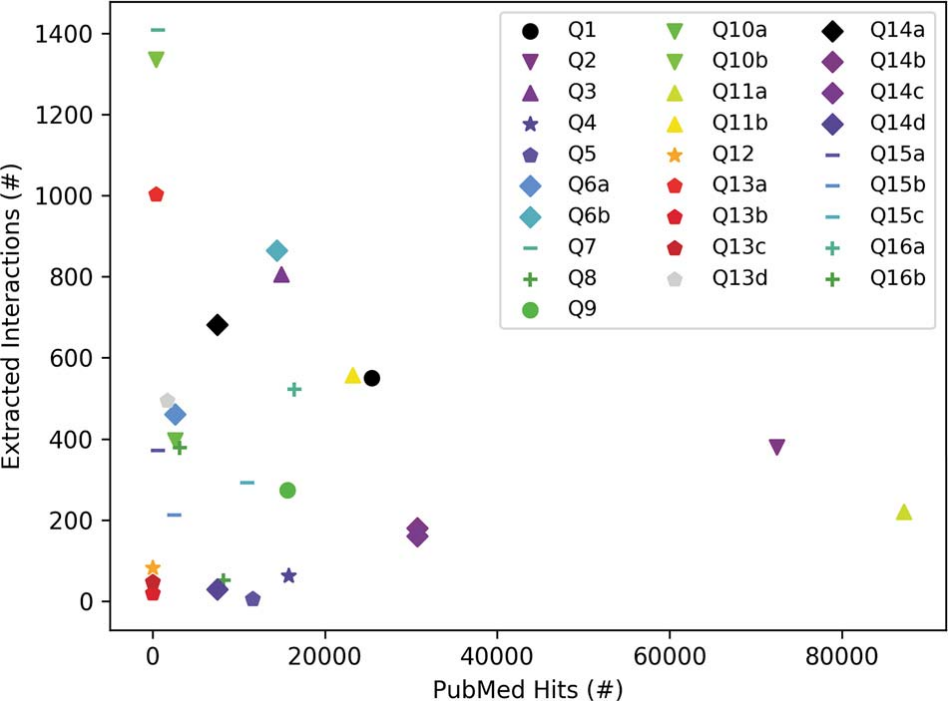
Influence of query category and term choice (the legend corresponds to query numbers in Table 3) on the number of papers found in PubMed and on the number of interactions extracted from the top 200 papers (except Q12, Q13b and Q13c, where PubMed returned less than 200 hits). Results obtained for the same query topic category, but different term aliases, or different example terms, are grouped together with the same marker shape and similar color.

The results in Figure 5, which were obtained for the list of queries in Table 3, suggest that the selection of a query topic, the choice of terms and characters in the query, and the terms’ presence in literature can all affect the number of papers retrieved from PubMed. As Figure 5 shows, the number of papers returned by a PubMed search can vary several orders of magnitude (from tens to hundreds of thousands) for different queries. For example, a well-studied term (e.g. EGFR, Q11a) will return many papers, whereas a recent discovery (e.g. copb2*, Q12) will have fewer PubMed hits. Furthermore, for terms with special characters or multiple aliases, machine reading may have difficulty extracting all relevant interactions, as shown by examples Q13a–d, where searches for different aliases of RSK90 all returned a different number of papers. To get comprehensive results, all well-known aliases may have to be included in a query. These results highlight the importance of the careful choice of query terms.

To explore the influence of query topic on the number of interactions that machine reading can extract, for each query term, we chose either the top 200 PubMed hits with valid PMC IDs (when the number of PubMed hits is larger than 200), or all the found papers (when the number of PubMed hits is smaller than 200). We chose 200 papers to ensure that every query would return at least a few dozen interactions. The results in Figure 5 suggest that the query topic and the choice of query terms could have a significant impact on the size of the machine reading output, as the number of extracted interactions does not seem to be correlated with the number of papers read. As expected, the query topic influences the selection of papers, while scientific texts can vary in the level at which they describe systems, from high-level review papers, to those that focus on precise mechanistic details of a small number of biochemical interactions. The choice of query terms, and the characters that are used in these terms can also have a strong influence, if machine readers are not trained to recognize most of the aliases of the same entity.

Interestingly, the selection of a query topic and query terms did not have a noticeable influence on the FLUTE output. In other words, while being conservative and selecting only 8.86% (mean computed for the 28 queries in Table 3) of the overall number of instructions provided by machine reading, this percent was relatively consistent across queries (standard deviation of 4.02%). These results suggest that FLUTE can reliably filter interactions for any query category, that is, it provides to model assembly only those interactions that have high confidence.

Finally, we also computed the speedup that FLUTE achieves, compared to manually filtering the interaction sets from the machine reading output. Assuming it would take a human approximately 30 seconds to judge one interaction, for each interaction set, we computed the average time for judging by a human, and we measured the FLUTE runtime. The average speedup that FLUTE achieved was 2560.28, with a standard deviation of 482.98, that is, FLUTE can increase the rate at which interactions are selected from hours to seconds or from days to minutes.

### Influence of interaction type

For the remainder of the study, we focused on three sets of interactions: the first two sets are obtained as a result of the two queries from Table 3, Q6b (Disease and Process query) and Q10b (Process and Protein query), and the third set (we will refer to it as a Multiple Protein query) is obtained using the REACH Explorer tool (6, 19) for several individual protein queries (MEK, ERK, AKT, GSK3, P70RSK, S6, CDK4, 4EBP1, YB1, SRC, CHK2, MTOR, and PI3K). For queries Q6b and Q10b, we read the 200 most relevant papers from PubMed, and REACH extracted 865 and 1336 interactions from these papers, respectively. In the third case, from the papers returned by the REACH Explorer tool, followed by the REACH Fetch tool (6) (when necessary to get more papers), followed by manual selection of ten relevant (all in the context of melanoma) papers for each of the 13 proteins. REACH read these 125 papers and extracted 6305 interactions.

**Figure 6:**
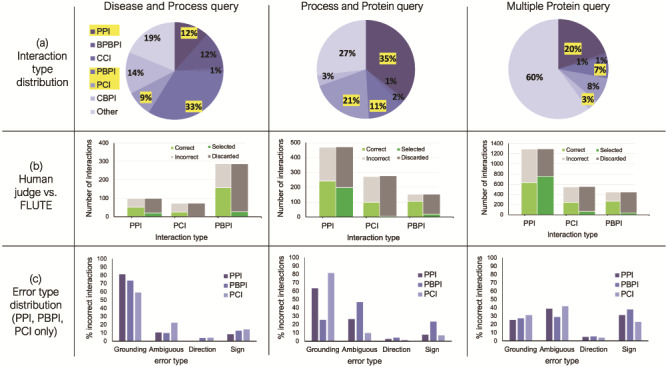
The influence of interaction type and machine reading errors on the number of selected interactions. (a) Overall distribution of interaction types for the three different queries, disease and biological process query, biological process and protein query and multiple protein query. (b) The comparison between FLUTE and manual selection; human judge decides whether interaction is correct given literature evidence, and FLUTE selects the interactions that are supported by databases. (c) The distribution of errors types in machine extraction of PPIs, PBPIs and PCIs for the three different queries.

To prepare the reading output for FLUTE, we determined the type for each interaction in these three sets, in particular, focusing on all the interactions where the interacting elements are either of protein (P), chemical (C), or biological process (BP) type (i.e. interactions of type PPI, PCI, PBPI, CCI, CBPI, and BPBPI), and all the other interactions are assigned to type Other (Figure 6a). The interaction type Other includes molecules such as mRNAs, protein families, or unknown types. Protein families are common, as well as complexes, however, these types are excluded from analysis in this work due to difficulty mapping to a standard identifier, and a lack of data on known interactions.

We processed the three sets of extracted interactions obtained from machine readers both manually and with FLUTE (Figure 6b). First, we assigned each interaction manually to one of the two groups, ‘correct’ and ‘incorrect’, based on whether the evidence statement that the machine reader provided agreed with the extracted interaction or not. We also used FLUTE to filter the same three sets of extracted interactions, that is, assign each interaction to either ‘selected’ or ‘discarded’ group, based on whether it was supported by the databases that FLUTE uses (as described in Methodology - Implementation of FLUTE).

The results shown in Figure 6b suggest that the accuracy of machine reading varies with different interaction types. From manual filtration, the PBPIs appear to be correct more often (54–69% correct), while the PCIs are the least likely to be correct (36–45% correct). Approximately half of all PPIs are correct (47–52% correct). Machine reading may erroneously extract PCIs from papers that use a recognized chemical in the methods protocol. Grounding may also be difficult for chemical compounds, due to the prevalence of non-standardized names. On the other end of the spectrum, PBPIs may be correct more frequently since biological process names are almost never abbreviated. Overall, for all three interaction sets, the number of correct interactions is approximately half the size of all the extracted non-other interactions. On the other hand, across all three sets of interactions, FLUTE selects much higher percent of PPIs, compared to the non-PPI interaction types. The number of interactions selected by FLUTE is also smaller than the number of interactions manually marked as ‘correct’. While the number of selected PPIs is similar to the number of correct PPIs, FLUTE is much less likely to select PCIs and PBPIs. This is due to the fact that the information on both PCIs and PBPIs is found less frequently in the databases used by FLUTE. This results in a much smaller output from FLUTE, compared to manual filtration, as well as a different distribution of interaction types in the final output.

### Influence of machine reading errors

To provide further guidance for the use of FLUTE, we investigated the types of errors in reading output, whether FLUTE is sensitive to the difference in machine reading error types, and also how well it can filter out the errors. Figure 6c shows the relative abundance of the four error types, Grounding, Omission, Direction, and Sign (see Methodology - The preprocessing of FLUTE inputs for definitions) in the three reading sets. For the Disease and Process query (Figure 6, left) and the Process and Protein query (Figure 6, middle), the distribution of error types varies slightly across different types of interactions (PPI, PBPI and PCI), with mostly Grounding and/or Omission errors across all three interaction types, while Sign errors are generally lower. For the Multiple Protein query (Figure 6, right), with the exception of Direction error, the other three error types remain consistent across interaction types. The machine reading output rarely had Direction errors in any query category or interaction type for the selection of manually curated interactions that we studied.

The results in Figure 6c suggest that FLUTE could be especially useful in the case of papers with proteins or genes that have non-standard names, or descriptions of complicated signaling pathways, such as those obtained for our example Disease and Process and Process and Protein queries. This is due to the fact that FLUTE is capable of filtering out a significant portion of interactions with Grounding and Omission errors. However, FLUTE does not address interactions with Direction or Sign errors, as STRING and STITCH do not always contain information about the direction and sign of interactions. Furthermore, GO annotations do not provide cause and effect information, only correlations, and therefore are not suitable for assignment of direction or sign.

Overall, FLUTE performed well on the interaction sets that we studied due to the relatively low occurrence of both Direction and Sign errors in these sets, but this may not be the case for other queries and interaction sets. In general, the information about direction and sign is critical for creating models that are used to study system dynamics, and a number of Direction and Sign errors can often be identified by examining contradictions within the machine reading output. In our future work, to be able to also filter out interactions with Direction or Sign errors, when the direction and sign information is not available in existing databases, FLUTE will be integrated with other tools designed to handle these error types and allow for manual expert input.

**Figure 7:**
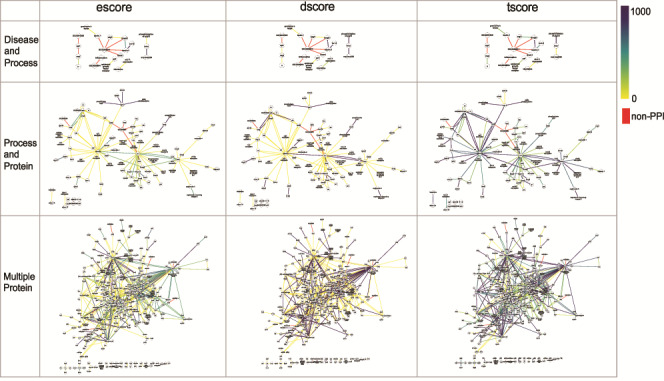
The networks of selected PPI interactions for the Disease and Process set (top row), Process and Protein set (middle row), and Multiple Protein set (bottom row), where each edge color represents a value of escore (left), dscore (middle) or tscore (right). Each PPI edge is colored by the indicated score type, from the minimum (0) to the maximum score (1000). A red edge indicates a non-PPI.

**Figure 8:**
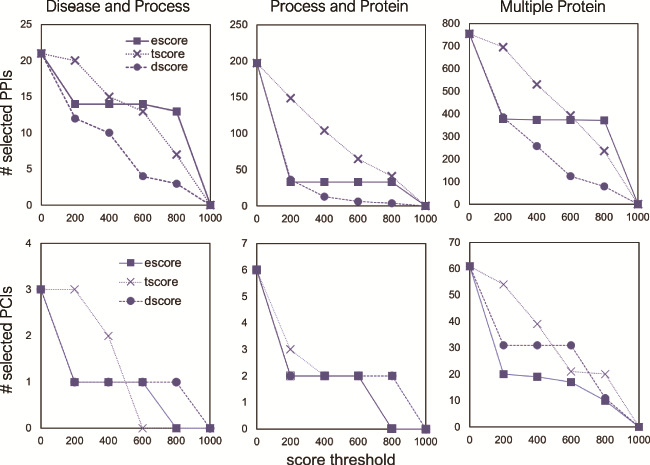
The number of selected interactions, PPIs (top) and PCIs (bottom) as a function of a score threshold for each score type, for the three different queries.

### Interaction scores and thresholds

FLUTE allows the user to choose confidence level for selected interactions, that is, for the three different score types (see Methodology - Implementation of FLUTE), the user can choose a score threshold value for interactions. This is best illustrated using the PPIs, due to the number of available interaction scores. Figure 7 highlights the diversity of different score types across the selected interactions for the Disease and Process, Process and Protein, and Multiple Protein interaction sets.

As can be seen from Figure 7, for a large set of selected interactions, the supporting evidence for the PPIs is wide-ranging. There may be tens to hundreds of papers used to determine escore and tscore, thus leading to many different score values present in the selected interactions. The escore and tscore are well-suited for assigning thresholds. Calculating the abstract co-mentions for the tscore is less onerous than interpreting biological evidence to calculate the escore. Therefore, choosing a score threshold for the escore, rather than the tscore, is more stringent. Pathway database resources are few and far between, and so the dscore is discrete, with only high or low values present in the output. To apply these findings to FLUTE threshold selection, the best practice may be to choose a tscore value as a threshold first, based on the size of the output desired. Results can be further refined by choosing an escore value as a threshold. In a small set of selected interactions (Figure 7, Disease and Process query results), the values for all three types of scores are much less diverse among the extracted interactions. With only a few nonzero score values, a small threshold may discard most of the present interactions. From these results, we find that FLUTE thresholding may not be very useful for small interaction sets.

Using several threshold values (0, 200, 400, 600, 800) for the three STRING score types, we examined the effect of score types and their values on the FLUTE output size. As Figure 8a shows, the number of selected PPIs decreases with the increase of a threshold. While the number of selected interactions decreases linearly with escore and tscore thresholds, the number of selected interactions is affected only at very low or very high threshold value for the dscore. The escore and dscore metrics are stringent due to the type of evidence required: either evidence of physical binding, or a well-known association present in a pathway database, respectively. The tscore seems to be least selective, allowing more interactions to pass through the filter, while escore causes the largest reduction of output size. Since the tscore is calculated using abstract co-mentions, we have less confidence in the results using a tscore threshold than if an escore threshold had been used.

**Figure 9:**
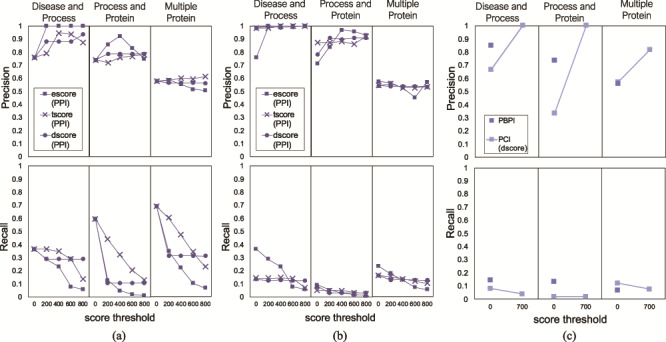
Precision and recall of FLUTE, compared to human judging, and the sensitivity of precision and recall to the scores, for the three different queries: (a) precision and recall when filtering PPIs with only one subscore at a time, (b) average precision and recall when filtering PPIs for all possible subscore combinations and (c) precision and recall when filtering PBPI and PCIs.

For PCIs, we can implement a similar threshold-based approach using the score types from the STITCH database. However, due to the scarcity of PCIs in the selected output, we are only able to use the STITCH escore as a threshold (Figure 8b). Similar to the PPIs, the number of selected PCIs decreases with the increase in the score threshold. Any escore threshold larger than 0, for all three queries, decreases the number of selected PCIs by ~66–67%. While the escore metric appears to be the most stringent for all the interaction sets that we used, those interactions that go through the filter using the escore have concrete evidence of physical interaction. Therefore, we have higher confidence in any interactions, either PPIs or PCIs, which are selected using the escore.

### FLUTE precision and recall

To validate the correctness of the PPIs selected by FLUTE, we compared the overlap between human judgment and FLUTE output. For each query, we calculated the percent of PPIs selected by FLUTE that were also marked as correct (precision), as well as the total number of interactions manually judged as correct that were also selected by FLUTE (recall). For each score type (escore, tscore, or dscore), we calculated the precision and recall at scores 0–1000, with intervals of 200. We tested both the effect of using one subscore as a threshold and using a combination of all three subscore types. Figure 9a shows the effect of changing one subscore threshold at a time while the other two subscores have no threshold constraints. In Figure 9b, the average precision and recall is calculated for each of the 125 different score type combinations. To get the average precision and recall, we took the mean for each 25 precision and recall values where one score type is kept with a constant value. In Figure 9c, we show precision and recall for PCIs at one threshold, due to the small output size of filtered PCIs, and PBPIs supported by the GO database. Using one subscore threshold at a time favors higher recall, at the cost of precision, while using multiple subscore thresholds together results in high precision but low recall. For the Multiple Protein interaction set, increasing the threshold did not increase precision; however, it did for the other two queries. Recall decreased in response to raising the score threshold, since higher thresholds exclude more interactions. As the threshold is increased, FLUTE inevitably excludes more correct interactions in the selected output.

**Table 4:** The effect of inclusion of between-paper duplicates or potentially novel interactions on precision and recall in PPIs

Query		Any STRING score ≥ 0	Any STRING score ≥ 0 or published after 2014	Any STRING score ≥ 0 or 2 + duplicates	Any STRING score ≥ 0 or 4 + duplicates	Any STRING score ≥ 0 or 6 + duplicates	Any STRING score ≥ 0 or 2 + duplicates or published after 2014
Process and protein	Precision	0.74	0.53	0.74	0.73	0.72	0.74
	Recall	0.60 (+0)	0.91 (+74)	0.66 (+15)	0.63 (+7)	0.62 (+6)	0.92 (+76)
Disease and process	Precision	0.76	0.37	—	—	—	—
	Recall	0.37 (+0)	0.48 (+6)	—	—	—	—
Multiple protein	Precision	0.58	0.57	0.57	0.58	0.58	0.55
	Recall	0.69 (+0)	0.75 (+38)	0.72 (+18)	0.70 (+9)	0.70 (+3)	0.77 (+50)

Numbers in parentheses beside the recall values indicate the number of true interactions added by using non-database filters.

**Table 5:** The effect of inclusion of between-paper duplicates or potentially novel interactions on precision and recall in PCIs

Query		Any STITCH score ≥ 0	Any STITCH score ≥ 0 or published after 2014	Any STITCH score ≥ 0 or 2 + duplicates
Process and protein	Precision	0.33	0.36	0.66
	Recall	0.02 (+0)	0.90 (+88)	0.43 (+40)
Disease and process	Precision	0.67	0.34	0.75
	Recall	0.08 (+0)	0.40 (+8)	0.12 (+1)
Multiple protein	Precision	0.54	0.43	0.48
	Recall	0.14 (+0)	0.29 (+36)	0.25 (+26)

Numbers in parentheses beside the recall values indicate the number of true interactions added by using non-database filters.

**Table 6:** The effect of inclusion of between-paper duplicates or potentially novel interactions on precision and recall in PBPIs

Query		Any GO annotation	Any GO annotation or published after 2014	Any GO annotation or 2 + duplicates
Process and protein	Precision	0.74	0.68	0.76
	Recall	0.13 (+0)	0.79 (+69)	0.51 (+40)
Disease and process	Precision	0.85	0.42	0.84
	Recall	0.18 (+0)	0.51 (+52)	0.39(+33)
Multiple protein	Precision	0.67	0.65	0.67
	Recall	0.13 (+0)	0.38 (+59)	0.34 (+56)

Numbers in parentheses beside the recall values indicate the number of true interactions added by using non-database filters.

The increase of precision in response to more stringent score thresholds (Figure 9) indicates that higher STRING and STITCH scores are correlated with correct machine reading output. Any of the three STRING score types that we tested, or the STITCH escore, are capable of differentiating between correct and incorrect machine reading output. Using interaction databases to inform interaction selection results in a higher-confidence output. Overall, the results in Figure 9 suggest that FLUTE can prioritize either ‘quality’ or ‘quantity’ of interactions, depending on user-determined thresholds. Selecting a low FLUTE threshold will output a higher quantity of interactions, at the cost of the correctness of the individual interactions. By comparison, a high threshold will output less interactions; however, we will have more confidence in the results.

Tables 4–6 show the updated precision and recall when different combinations of database and non-database thresholds are used, for filtering PPIs, PCIs and PBPIs. We chose the publication year threshold based on when the oldest dataset was gathered in the three interaction sets, Disease and Process, Process and Protein, and Multiple Protein. The Multiple Protein interactions set was obtained from papers published as recently as 2016, while the other two sets were obtained from papers up until 2018. Therefore, for the PPIs (Table 4), we chose interactions published after 2014, which would return potentially novel interactions. Next, we chose either 2, 4 or 6 duplicates (interactions extracted from 2, 4 or 6 papers, respectively) as anything beyond 6+ duplicates is extremely rare in our interaction set. For the Disease and Process PPI dataset, the output is small enough that there are no between-paper duplicates. The upper limit on number of duplicates increases as the size of the interaction set increases, so the optimal non-database thresholds change for each interaction set. As described in Methodology - Implementation of FLUTE, we flag these interactions, and add them to the PPIs filtered using the FLUTE database thresholds. We similarly filter the PCIs (Table 5) and PBPIs (Table 6), using the least stringent interaction database threshold, a 2014 publication year thresholds, or a thresholds of at least two between-paper duplicates. As expected, interactions selected using these non-database filters greatly increase the recall of FLUTE output; however, this comes at a cost to precision. These results show that using flags may be useful for indicating interactions that could benefit from manual review, but these thresholds are not rigorous enough to warrant automatic inclusion into filtered output. The research topic determines the queries as well as the machine reading output sets, and therefore, the optimal combination of thresholds will be largely context dependent.

**Figure 10:**
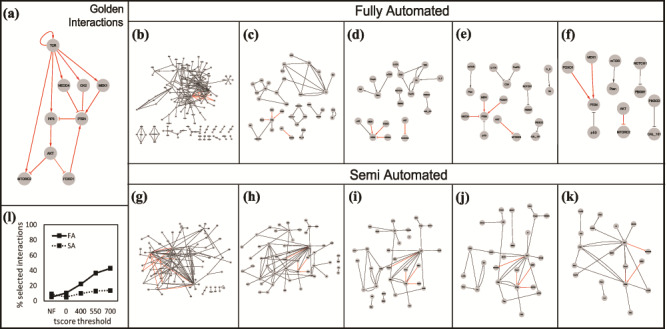
(a) The golden set of interactions from (27). The gray box in the right-hand corner shows the number of golden interactions present in each set in red. Interactions obtained using the Fully Automated (FA) approach: (b) unfiltered, (c) filtered without thresholds, (d) tscore > 400, (e) tscore > 550, (f) tscore > 700. Interactions obtained using the Semi Automated (SA) approach: (g) unfiltered, (h) filtered, no thresholds, (i) tscore > 400, (j) tscore > 550, (k) tscore > 700. (l) Comparison of the two approaches: % of selected interactions in each approach as a function of a tscore threshold.

### FLUTE database-based expansion of interaction set

We used the FLUTE database to supplement the machine-extracted PPIs for our three queries. While additional interactions cited in the same papers were identified, the output is very sparse. In Table 1 (Supplement), we see that there are very few interactions from these citations that are in the FLUTE database. This can be explained by the small overlap between the papers cited in the reading sets and the papers already added to databases. If users want to supplement machine reading output, the interactions output by FLUTE using this approach may not be directed, so manual review is necessary to obtain this information.

For the FLUTE paper retrieval utility, we show four example queries that include proteins Raf1, EGFR, RelA, and Copb2 in Table 2 (Supplement). We show PubMed hits to provide a frame of reference for how well-studied an interaction is. Our first three examples are well established proteins, with hundreds or thousands of PubMed papers. Our last example (Copb2) has only 37 relevant papers in PubMed. It is important to note that we queried PubMed with the exact query provided in the first column, which could explain why we found more papers in the FLUTE database than PubMed. We use the UniProt ID for querying the FLUTE database, which could identify more papers than using the gene name in PubMed. Using FLUTE’s new paper retrieval utility, we located tens to hundreds of papers for each protein. This number would be much larger if we used all protein-protein interaction databases. For now, we use only BioGrid and Reactome, which are much smaller and more manageable than STRING. Using these two databases, we are able to find many papers for machine reading, without prohibitively costly runtimes (on the scale of seconds to minutes, rather than hours).

**Figure 11:**
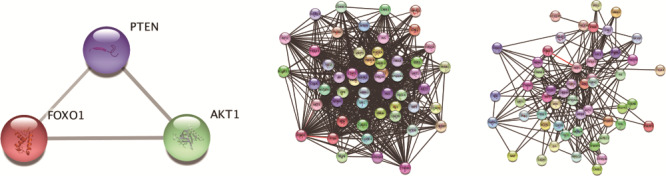
Results of STRING search for the T-cell case study: (a) Interactions between the STRING search terms (PTEN, AKT1 and FOXO1) illustrated as a graph. (b) Expanded network with 50 additional nodes, threshold of 0.65. (c) Expanded network with 50 additional nodes, threshold of 0.95. (NOTE: Unlike the STRING database, the STRING web application uses score values within the [0,1] interval, where 0 is low-confidence, and 1 is high confidence. Interactions from the list of golden interactions illustrated in Figure 10a are in red.)

### Case study

We used a previously published model of the circuitry that controls T-cell differentiation (27) to show that FLUTE can indeed improve the accuracy and the relevance of machine reading. Our goal is to automatically retrieve from literature the ‘golden’ set of interactions, illustrated as a graph in Figure 10a. First, we created a literature search query to obtain relevant papers from PubMed (equation (1)).

Next, using REACH, we obtained interactions from the top 100 papers (returned as a result of PubMed search), as well as interactions from all the citations (29 references) of the model in (27) that include T cell signaling studies. We refer to these two sets of extracted interactions as fully automated reading set (FA) and semi-automated reading set (SA), respectively. The interactions from the FA and SA reading sets can be seen in Figure 10b and Figure 10g. For both reading sets, many more interactions than simply the desired set are extracted. Among the 264 interactions from the FA reading set, only 14 (the golden set) are relevant to our desired results. Similarly, only 14 of the 154 interactions from the SA reading set are relevant. To demonstrate the usefulness of FLUTE, we show the results of filtering the reading sets with and without setting score thresholds (Figure 10b–k).

As already demonstrated in Results-Interaction scores and thresholds, increasing the threshold decreases the size of the FLUTE output. We chose several thresholds to test whether FLUTE was capable of discarding irrelevant interactions, as well as selecting the desired interactions. Before filtration, the desired interactions only composed 5–9% of the total reading output. By using several tscore thresholds (Figure 10d–f,i–k), the percent of relevant interactions in the FLUTE output set can be increased to 22–43%. FLUTE removes a large number of incorrect interactions from the overall interaction set, while still retaining a significant portion of the desired interactions. Therefore, the percentage of relevant interactions is much higher in the FLUTE output than in the original sets, and this is especially notable in the larger FA reading set.

To demonstrate the difference between FLUTE and the approach that uses only publicly available interaction databases, we also retrieved PPIs using the ‘Multiple proteins’ search feature in the STRING web application (12). Similar to the FA exercise described above, we included PTEN, AKT1, and FOXO1 as search terms for the STRING web application (Figure 11). Since cell type-specific results are unavailable in STRING, we were unable to add a T-cell-specific search term. We then expanded the network using the interactions suggested by STRING for two thresholds of the combined score (detailed in (10)). The combined score is a metric that takes into account both the individual scores, and the probability of randomly observing an interaction. Unlike the thresholds used in the FLUTE methodology, the STRING web application uses a threshold for the combined score only, and not for individual scores. We chose two thresholds, medium and high (Figure 11a,b), to explore the effect of the combined score threshold on the interaction set in STRING output. We decided to limit the number of additional nodes that are selected through the STRING search to 50, in order to retrieve a large enough number of interactions for expanding the model, but still manageable for human judgment. Less than 0.5% of both outputs were relevant, regardless of the score threshold. Figure 11 shows that even through selecting only high-confidence interactions by increasing the threshold, we are unable to recreate a well-established interaction network. The confidence in interactions retrieved from public databases like STRING is not the issue, but the relevance of the interactions. Contextual search terms, such as cell-type or disease state, are needed to locate specific interaction sets. These results show that simply using an interaction database is unable to capture the same number of golden interactions, and also contributes dozens of non-relevant interactions. By combining the strengths of machine reading and interaction databases, FLUTE is successful at selecting believable, relevant interactions.

## Conclusion

The process of model assembly is tedious and time consuming, requiring hundreds of hours of reading literature to generate one model. By automating steps in this process, high accuracy models can be generated rapidly. For frameworks that use NLP methods to extract potential model elements and interactions from literature, a filtration method can be used to guarantee that only high-confidence interactions are added to the model. Our proposed filtering tool, FLUTE, enables this selection using publicly available data.

FLUTE not only decreases the number of interactions that need to be tested for model improvement, it also keeps only the high-quality interactions. FLUTE eliminates misinterpreted or low-confidence interactions. This reduces the amount of work needed for creating models both manually and automatically and ensures that models assembled automatically rely on biologically accurate data.

While FLUTE is capable of returning high-quality interactions, it also discards accurate interactions depending on the threshold used. Although the optional thresholds for between-paper duplicates and for recent publications increase the recall of correct interactions, these thresholds are highly context-specific, and precision is penalized in some cases. These literature-based filters can help further reduce the time needed for manual review of interactions, but they do not fully eliminate the necessity for human intervention. To accommodate novel machine reading results that are accurate, additional features that draw from NLP can be added, such as trigger words, or analyzing sentence structure. These features could help to judge the quality of the reading output that would complement databases with historical information, and they could provide further insight into the reliability of individual interactions without penalizing novel interactions. We plan to explore these directions to extend FLUTE functionality in the future.

To better address sign errors from phosphorylation events, we can incorporate data from sources such as PhosphoSitePlus (28), which curates information on the effect of a phosphorylation event. Instead of a single database for each different interaction type, we will explore use of composite network databases such as NDEx (29). To include interactions between protein families and other elements, databases such as PFAM (30) could be incorporated. This would allow for the inclusion of more abstract interactions that describe general trends in signaling.

The FLUTE function that retrieves interactions from the FLUTE database that cite papers in our reading set, outputs many undirected interactions. In order to use these interactions by tools that automatically assemble and extend executable models (31–33), the information about the directionality and sign of these interactions is important, and our future work will focus on locating relevant, directed interactions. In addition to retrieving interactions from the FLUTE database, we developed another FLUTE function that utilizes this database to find papers for machine reading, and thus, streamlines another step in the machine reading workflow, by ensuring that all papers used for machine reading contain cell signaling events.

Other future directions include expansion of the database used to validate the accuracy of NLP results to improve the paper retrieval utility, as well as incorporating STRING publication data and exploring ways to speed up the literature search process.

## Supplementary Material

SupplementaryTable1

SupplementaryTable2
